# Differential effects of *KRAS* mutational status on long-term survival according to the timing of colorectal liver metastases

**DOI:** 10.1186/s12885-021-08144-5

**Published:** 2021-04-15

**Authors:** Nozomu Sakai, Katsunori Furukawa, Tsukasa Takayashiki, Satoshi Kuboki, Shigetsugu Takano, Masayuki Ohtsuka

**Affiliations:** Department of General Surgery, Graduate School of Medicine, Chiba University, 1-8-1 Inohana, Chuo-ku, Chiba, 260-8670 Japan

**Keywords:** Colorectal cancer, Liver metastases, *KRAS*, Synchronous metastasis, Hepatectomy

## Abstract

**Background:**

The relationship between *KRAS* mutational status and timing of colorectal liver metastasis (CRLM) remains unclear. This study evaluated the relationship between *KRAS* mutational status and long-term survival in patients with synchronous CRLM.

**Methods:**

Of the 255 patients who underwent initial hepatic resection for CRLM between January 2001 and December 2018, the *KRAS* mutational status was examined in 101 patients. Medical records of these patients were reviewed to evaluate recurrence and survival outcomes.

**Results:**

*KRAS* mutant status was identified in 38 patients (37.6%). The overall survival (OS) was significantly better in patients with wild-type *KRAS* than in those with mutant *KRAS* status. In patients with synchronous metastases, the OS of patients with wild-type *KRAS* was significantly better than those with mutant *KRAS*. Multivariate analyses indicated shorter OS to be independently associated with positive primary lymph node, and large tumor size and R1 resection in patients with metachronous metastasis, whereas to be independently associated with mutant *KRAS* status in patients with synchronous metastasis. Furthermore, in the subgroup of patients with synchronous metastases, the repeat resection rate for hepatic recurrence was significantly high in those with wild type *KRAS* than in those with mutant *KRAS*.

**Conclusion:**

*KRAS* mutation is an independent prognostic factor in patients with synchronous CRLM, but not in patients with metachronous CRLM.

## Introduction

Colorectal cancer (CRC) is one of the common causes of cancer-related mortality worldwide. Distant metastasis is strongly associated with poor prognosis in patients with CRC. During the course of CRC, colorectal liver metastases (CRLM) occur in approximately half of the patients [1]. Surgical resection is the primary treatment modality for CRLM, which ensures complete restoration or long-term survival in the patients. The 5-year OS rate in patients with CRLM after surgical resection is currently 33–51% [1–3]. Historically, hepatic resection for CRLM was indicated for tumors isolated only in the liver with less aggressive features (i.e., low number, small size) [4]. However, the indication for hepatic resection for CRLM has been extended over the past decades to include more patients with aggressive disease features. Recent studies demonstrated that the presence of extrahepatic metastases is no longer a contraindication for surgical resection in patients with colorectal metastases [5–7]. In the past, one centimeter was considered an adequate surgical margin [8, 9]. However, recent data have shown that a 1 cm margin is not a requisite for curative resection, and that margin width does not affect long-term survival [10, 11]. One reason for these changes might be attributable to the use of perioperative chemotherapy with molecular targeted agents [12]. In fact, systemic chemotherapy has been evolving since the late 2000s, especially after the EPOC study [13]. Moreover, several randomized control trials demonstrated the clinical implication of KRAS mutational status and RAS mutational status is now commonly used as a determinant of anti-EGFR antibody administration in modern chemotherapy regimens [14, 15].

Synchronous metastasis is associated with shorter disease-free survival duration and may correlate with the more disseminated disease than that with metachronous metastasis [16]. A recent nationwide survey in Japan demonstrated that adjuvant chemotherapy is associated with a favorable prognosis in patients with synchronous CRLM, but not in those with metachronous CRLM [17]. Another study suggested that adjuvant chemotherapy is more effective in cases with synchronous metastases than in those with metachronous metastases [18]. These data highlight the differences in tumor biology between synchronous and metachronous metastases. Recent studies have revealed that somatic mutations in genes such as *KRAS* and *BRAF* are associated with poor clinical outcomes in patients with CRLM [19–24]. Mutations in *KRAS* are found in about 30% of the patients with CRLM [20]. However, fewer studies have studied the association between the mutational status of *KRAS* and the timing of CRLM.

The aim of the present study was to evaluate the relationship between the mutational status of *KRAS* and long-term survival in patients with synchronous CRLM.

## Patients and methods

### Patients

A total of 255 patients underwent initial hepatic resection for CRLM at the Department of General Surgery, Chiba University, between January 2001 and December 2018. Of these, the mutation status of *KRAS* was examined in 101 patients. The medical records of these consecutive patients were reviewed retrospectively. This study was approved by the Institutional Ethics Committee of the College of Medicine, Chiba University, Japan. Informed consent was obtained from all the patients after explaining the extent of the disease, and the benefits and risks associated with the treatments.

### Surgical procedure

Pringle’s maneuver was used, whenever possible, to decrease intraoperative bleeding from the cut surface of the liver. Transection of the liver parenchyma was performed in all patients using a cavitron ultrasonic surgical aspirator.

### KRAS mutation profiling

DNA was extracted from the paraffin blocks of primary CRC or CRLM. Polymerase chain reaction (PCR)-based primer extension assay was performed to screen for genomic mutations encoding residues 12 and 13 of the KRAS protein.

### Definition of synchronous metastases

Synchronous metastases was defined as the metastases that are clinically and/or radiologically detected when the primary cancer is diagnosed.

### Definition of surgical margin

All the resected specimens were subjected to a routine pathological examination. The cases with R1 resection were identified based on microscopically incomplete resection with the presence of tumor invasion on the cut surface (i.e., a tumor-free margin of 0 mm).

### Statistical analysis

All data were retrospectively collected and differences were considered statistically significant at *P*-values < 0.05. Relationships between categorical variables were assessed using either the chi-square test or Fisher’s exact test, as appropriate. Survival outcomes after the initial hepatectomy for CRLM were evaluated using the Kaplan-Meier method and log-rank test. Survival data were evaluated using univariate and multivariate Cox proportional regression analyses. All the statistical analyses were performed using the JMP Pro software (version 13; SAS Institute Japan, Tokyo, Japan).

## Results

### Patients’ characteristics and perioperative data

The demographic and clinicopathological characteristics of patients are summarized in Table 1. Of the 101 patients, 63 (62.4%) had wild-type *KRAS* status (*KRAS*-wt), whereas 38 (37.6%) had mutant *KRAS* status (*KRAS*-mut). As indicated in Table 1, there were no significant differences in patient characteristics based on the mutational status of *KRAS*.

**Table 1:** Patient characteristics

Characteristics			*P*-value
	*KRAS*-wt (*n* = 63) n (%)	*KRAS*-mut (*n* = 38) n (%)	
Sex, male/female	36/27 (57.1/42.9)	22/16 (57.9/42.1)	1.000
Median age (range) (years)	66 (33–83)	69 (35–82)	0.821
Primary tumor			
Right-sided/Left-sided/rectum	11/31/21 (17.5/49.2/33.3)	7/18/13 (18.4/47.4/34.2)	0.983
pT1–3 / T4	36/27 (57.1/42.9)	23/15 (60.5/39.5)	0.738
Node positive / node negative	48/15 (76.2/23.8)	30/8 (78.9/21.1)	0.811
Initial liver metastases			
Synchronous / metachronous diagnosis	34/29 (54.0/46.0)	22/16 (57.9/42.1)	0.837
Unilobar / bilobar	36/27 (57.1/42.9)	18/20 (47.4/52.6)	0.412
Mean number of tumors, (range)	3.7 (1–26)	3.6 (1–19)	0.910
Solitary / multiple metastases	23/40 (36.5/63.5)	12/26 (31.6/68.4)	0.670
Largest tumor’s mean size, cm,	3.8 (0.5–13)	3.5 (0.8–10)	0.531
Serum carcinoembryonic antigen (ng/mL), < 5 / ≥5	17/46 (27.0/73.0)	13/25 (34.2/65.8)	0.503
Hepatectomy, minor/major	43/20 (68.3/31.8)	27/11 (71.1/28.9)	0.827
Surgical margin, R0/R1	44/19 (69.8/30.2)	26/12 (68.4/31.6)	1.000
Preoperative chemotherapy, yes/no	37/26 (58.7/41.3)	23/15 (60.5/39.5)	1.000
Adjuvant chemotherapy, yes/no	41/22 (65.1/34.9)	25/13 (65.8/34.2)	1.000

Values are number of patients or mean/median values, as indicated

### Oncological outcomes

**Fig. 1:**
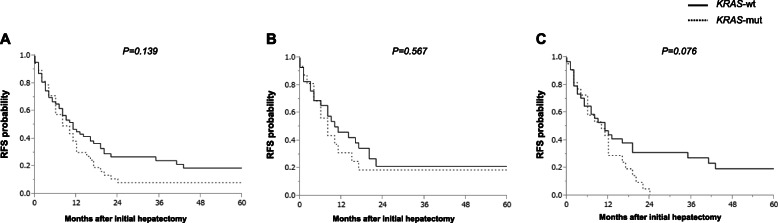
Kaplan-Meier survival curves for recurrence-free survival. **a** RFS according to the *KRAS* mutational status in patients with colorectal liver metastases (CLRM). Subgroup analyses for RFS in patients with (**b**) metachronous and (**c**) synchronous metastases. RFS, recurrence-free survival

**Fig. 2:**
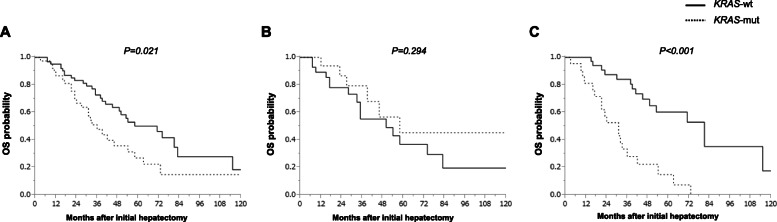
Kaplan-Meier survival curves for overall survival. **a** OS according to the *KRAS* mutational status in patients with colorectal liver metastases (CLRM). Subgroup analyses for OS in patients with (**b**) metachronous and (**c**) synchronous metastases. OS, overall survival

The 3-year recurrence-free survival (RFS) and 5-year OS rates were 14.9 and 41.2%, respectively, for all the patients. In *KRAS*-wt patients, the median RFS was 11 months and the 3-year RFS rate was 24.2%. In *KRAS*-mut patients, the median RFS was 8 months and the 3-year RFS rate was 8.2% (Fig. 1a). Further, in *KRAS*-wt patients, the median OS was 71 months and the 5-year OS rate was 50.1%. In *KRAS*-mut patients, the median OS was 36 months and the 5-year OS rate was 26.8% (Fig. 2a). There was no significant difference between RFS for *KRAS*-wt and *KRAS*-mut patients (*P* = 0.139). Whereas the OS rate was found to be significantly better in patients with *KRAS*-wt than in those with *KRAS*-mut status (*P* = 0.021). Next, in the subgroup analysis, RFS was not significantly different based on the *KRAS* mutational status in patients with metachronous metastases (3-year RFS rates and median RFS: 21.5% and 11 months in *KRAS*-wt, while 18.8% and 10 months in *KRAS*-mut patients, respectively; *P* = 0.567) (Fig. 1b). In patients with synchronous metastases, the RFS of *KRAS*-wt patients was relatively better, but not significant, than those with *KRAS*-mut status (3-year RFS rate and median RFS: 27.4% and 11 months in patients with *KRAS*-wt, and 0.0% and 10 months in those with *KRAS*-mut status, respectively; *P* = 0.076) (Fig. 1c). The OS was not significantly different based on the *KRAS* mutational status in patients with metachronous metastases (5-year OS rate and median OS: 36.8% and 50 months in *KRAS*-wt versus 45.3% and 58 months in *KRAS*-mut; *P* = 0.294) (Fig. 2b). Whereas, the OS of patients with synchronous metastases harboring *KRAS*-wt was significantly better than those harboring *KRAS*-mut status (5-year OS rate and median OS: 60.4% and 81 months in *KRAS*-wt versus 14.9% and 31 months in *KRAS*-mut; *P* < 0.001) (Fig. 2c). Furthermore, the univariate analyses revealed that shorter OS duration in patients was associated with synchronous metastasis (*P* = 0.050), bilobar tumor distribution (*P* = 0.005), increased number of tumors (*P* = 0.001), large tumor size (≥ 5 cm, *P* < 0.001), high serum carcinoembryonic antigen levels (≥5 ng/mL, *P* = 0.029), major hepatectomy (*P* = 0.002), R1 resection (*P* = 0.002), and mutant KRAS (*P* = 0.026) (Table 2). In multivariate analyses, shorter OS duration in patients was found to independently associate with R1 resection (hazard ratio [HR]: 2.554, *P* = 0.003), and mutant *KRAS* status (HR: 2.409, *P* = 0.003) (Table 2). In patients with metachronous metastasis, shorter OS duration was found to be independently associated with positive primary lymph node (HR: 1.779, *P* = 0.039), large tumor size (≥5 cm, HR: 3.010, *P* < 0.001), and R1 resection (HR: 2.060, *P* = 0.021) (Table 3). In patients with synchronous metastasis, shorter OS duration was found to independently associate with mutant *KRAS* status (HR: 4.316, *P* < 0.001) (Table 4).

**Table 2:** Predictive factors of shorter OS (*n* = 101)

Variables	Univariate analysis		Multivariate analysis		
	HR	*P*-value	HR	95% CI	*P*-value
Sex, male	1.209	0.282			
Age	1.007	0.344			
Primary tumor					
Location, colon/rectum	0.888	0.503			
Location, right-sided/left-sided	0.818	0.385			
pT stage	1.274	0.092			
Positive lymph node	1.426	0.062			
Initial liver metastases					
Timing of diagnosis: synchronous	1.405	0.050	1.186	0.669–2.144	0.562
Tumor distribution: bilobar	1.642	0.005	1.106	0.594–2.103	0.752
No. of tumors	1.072	0.001			
No. of tumors (solitary/multiple): multiple	2.050	< 0.001	1.010	0.523–2.436	0.807
Tumor size ≥5 cm	2.122	< 0.001	1.092	0.541–2.125	0.801
Serum carcinoembryonic antigen ≥5 ng/mL	1.586	0.029	1.964	0.991–4.188	0.053
Type of hepatectomy: major	1.812	0.002	1.415	0.762–2.586	0.268
Surgical margin: R1	1.805	0.002	2.554	1.385–4.750	0.003
*KRAS* mutational status: *KRAS*-mut	1.860	0.026	2.409	1.361–4.250	0.003

*HR* Hazard ratio; *CI* Confidence interval

**Table 3:** Predictive factors of shorter OS (metachronous) (*n* = 45)

Variables	Univariate analysis		Multivariate analysis		
	HR	*P*-value	HR	95% CI	*P*-value
Sex, male	1.060	0.824			
Age	1.119	0.854			
Primary tumor					
Location, colon/rectum	1.035	0.899			
Location, right-sided/left-sided	0.784	0.473			
pT stage	1.204	0.350			
Positive lymph node	1.756	0.042	1.779	1.028–3.223	0.039
Initial liver metastases					
Tumor distribution: bilobar	1.256	0.420			
No. of tumors	1.053	0.127			
No. of tumors (solitary/multiple): multiple	1.489	0.123			
Tumor size ≥5 cm	3.023	< 0.001	3.010	1.734–5.131	< 0.001
Serum carcinoembryonic antigen ≥5 ng/mL	2.075	0.023	1.701	0.882–3.612	0.117
Type of hepatectomy: major	1.377	0.328			
Surgical margin: R1	1.898	0.034	2.060	1.122–3.632	0.021
*KRAS* mutational status: *KRAS*-mut	0.608	0.289			

*HR* Hazard ratio; *CI* Confidence interval

**Table 4:** Predictive factors of shorter OS (synchronous) (*n* = 56)

Variable	Univariate analysis		Multivariate analysis		
	HR	*P*-value	HR	95% CI	*P*-value
Sex, male	1.421	0.139			
Age	1.016	0.133			
Primary tumor					
Location, colon/rectum	0.783	0.303			
Location, right-sided/left-sided	0.818	0.529			
pT stage	1.232	0.344			
Positive lymph node	1.083	0.765			
Initial liver metastases					
Tumor distribution: bilobar	1.857	0.009	1.312	0.562–3.138	0.530
No. of tumors	1.081	0.006	1.063	0.961–1.164	0.223
No. of tumors (solitary/multiple): multiple	2.930	< 0.001	0.920	0.298–3.230	0.890
Tumor size ≥5 cm	1.511	0.117			
Serum carcinoembryonic antigen ≥5 ng/mL	1.205	0.514			
Type of hepatectomy: major	1.976	0.005	1.221	0.535–2.765	0.632
Surgical margin: R1	1.641	0.047	1.718	0.740–3.887	0.202
*KRAS* mutational status: *KRAS*-mut	4.517	< 0.001	4.316	1.973–9.845	< 0.001

*HR* Hazard ratio; *CI* Confidence interval

### Recurrence after initial hepatectomy

Disease recurrence after the initial hepatectomy was observed in 82 patients (81.2%). There was an insignificant difference in recurrence rates between patients with *KRAS*-wt and *KRAS*-mut status (77.8% vs. 86.8%) (Table 5). Repeat resection was performed in 25 (51.0%) and 15 (45.5%) patients with *KRAS*-wt and *KRAS*-mut status, respectively (*P* = 0.658) (Table 6). In the subgroup of synchronous metastases, the repeat resection rate for all recurrence was 57.7% in patients with *KRAS*-wt and 30.0% in those with *KRAS*-mut status (*P* = 0.079) (Table 6). Hepatic recurrence was observed in 26 (41.3%) and 23 (60.5%) patients with *KRAS*-wt and *KRAS*-mut status, respectively (*P* = 0.068) (Table 7). Repeat hepatectomy was performed in 15 (57.7%) and 8 (34.8%) patients with *KRAS*-wt and *KRAS*-mut status, respectively (*P* = 0.154) (Table 8). Whereas, in the subgroup of synchronous metastases, the repeat resection rate for hepatic recurrence was 66.7% in patients with *KRAS*-wt and 14.3% in those with *KRAS*-mut status (*P* = 0.008) (Table 8). Repeat hepatectomy was not recommended in patients with synchronous metastases harboring *KRAS*-mut status due to multiple hepatic recurrence in 3 patients, and both hepatic and extrahepatic recurrence to the lung, peritoneal dissemination and bone in 9 patients. Of these, 9 patients developed recurrence within 12 months after initial hepatectomy. Moreover, extrahepatic recurrence (including both extrahepatic and hepatic recurrences) was observed in 18 (68.2%) and 15 patients (52.9%) with synchronous metastases harboring *KRAS*-wt and *KRAS*-mut status, respectively (*P* = 0.282).

**Table 5:** Recurrence after initial hepatectomy

	*KRAS*-wt *n* = 63	*KRAS*-mut *n* = 38	*P*-value
Total cohort	49/63 (77.8%)	33/38 (86.8%)	0.304
Synchronous metastases	26/34 (76.5%)	20/22 (90.9%)	0.285
Metachronous metastases	23/29 (79.3%)	13/16 (81.3%)	1.000

**Table 6:** Repeat resection for all recurrence after initial hepatectomy

	*KRAS*-wt *n* = 49	*KRAS*-mut *n* = 33	*P*-value
Total cohort	25/49 (51.0%)	15/33 (45.5%)	0.658
Synchronous metastases	15/26 (57.7%)	6/20 (30.0%)	0.079
Metachronous metastases	10/23 (43.5%)	9/13 (69.2%)	0.177

**Table 7:** Hepatic recurrence after initial hepatectomy

	*KRAS*-wt *n* = 63	*KRAS*-mut *n* = 38	*P*-value
Total cohort	26/63 (41.3%)	23/38 (60.5%)	0.068
Synchronous metastases	15/34 (44.1%)	14/22 (63.6%)	0.180
Metachronous metastases	11/29 (37.9%)	9/16 (56.3%)	0.348

**Table 8:** Repeat hepatectomy for hepatic recurrence after initial hepatectomy

	*KRAS*-wt *n* = 26	*KRAS*-mut *n* = 23	*P*-value
Total cohort	15/26 (57.7%)	8/23 (34.8%)	0.154
Synchronous metastases	10/15 (66.7%)	2/14 (14.3%)	0.008
Metachronous metastases	5/11 (45.5%)	6/9 (66.7%)	0.406

## Discussion

We aimed to delineate the relationship between *KRAS* mutational status and long-term survival in patients with CRLM, and assess whether there were any differences in the effect of *KRAS* mutational status on the disease outcome based on the timing of metastasis. The present study clearly demonstrated that the effect of the mutation status of *KRAS* varied according to the timing of liver metastasis. The *KRAS*-mut status was significantly associated with poor prognosis in patients with synchronous metastasis, but not in those with metachronous metastasis.

The reason for poor prognosis in patients with synchronous metastasis and *KRAS*-mut tumors may be partially explained by the recurrence patterns after initial hepatectomy. As reported previously, repeat hepatectomy for hepatic recurrence can yield survival benefit similar to that after initial hepatectomy [25]. Therefore, tumor recurrence itself is not always associated with poor prognosis, although unresectable recurrence is considered a distinct poor prognostic factor [26–28]. We found that the rate of re-resection for hepatic recurrence was significantly low in patients with synchronous metastases and *KRAS*-mut tumors, which might lead to poor prognosis in these patients than in those with synchronous metastases and *KRAS*-wt tumors.

Moreover, the rate of extrahepatic recurrence was relatively high in patients with synchronous metastases and *KRAS*-mut tumors than in those with synchronous metastases and *KRAS*-wt tumors. Since extrahepatic metastasis is also known to be associated with poor prognosis [6], this trend may affect the poor prognosis in patients.

Another reason could be the difference in response to systemic chemotherapy according to the *KRAS* mutation status. In the context of systemic chemotherapy, Mise et al. investigated the association between *RAS* mutational status and response to preoperative chemotherapy in patients with CRLM. They revealed that *RAS* mutations were significantly associated with minor pathological and suboptimal morphological responses, which were assessed using computed tomographic scans [29]. Other studies also demonstrated that *KRAS* mutations were significantly associated with minor response to chemotherapy in patients with CRLM, and that *RAS* mutation status may serve as a biomarker for response to chemotherapy [29–31]. In the present study, the effect of perioperative chemotherapy was not assessed since various treatment regimens were used during the extended study period. However, preoperative and adjuvant chemotherapy were administered to almost 60% of the patients. Therefore, the variable responses to chemotherapy may lead to prognostic differences observed in the present study. Moreover, about 70% of the patients with synchronous metastases and *KRAS*-mut tumors received perioperative chemotherapy. Despite the high rate of administering chemotherapy, a poor prognosis was observed in this subgroup, suggesting that unfavorable response to chemotherapy may have resulted in disseminated and/or unresectable recurrences.

Other than the *KRAS* mutation status, the univariate and multivariate analyses revealed several differences between synchronous and metachronous metastases in terms of clinicopathological factors associated with patients’ prognoses. In the context of the timing of metastasis, Tsai et al. demonstrated that synchronous metastasis was significantly associated with low disease-free survival, and proposed that it may represent a highly disseminated disease than that with metachronous metastasis [16]. Additionally, other studies have demonstrated that response to chemotherapy may vary with the timing of metastasis [18, 32]. Together, these results support the hypothesis that tumor biology may be influenced by the timing of metastasis. However, further studies would be required to ascertain the role of mutational statuses influencing the biological differences according to the timing of metastases.

Here, surgical margin status was not found to be a prognostic factor of synchronous metastasis. The clinical significance of surgical margin has been debated in past decades. Moreover, it has been reported that the extent of optimal tumor-free margin may vary according to the *KRAS* mutation status [33], with 1–4 mm margins being considered sufficient in patients with wild-type *KRAS*, whereas 1-cm margins being considered insufficient for those with mutant *KRAS* status. Furthermore, other studies reported that the *KRAS* mutation status was associated with a narrow width of surgical margin [34, 35]. We defined R1 resection as microscopically incomplete resection with the presence of tumor invasion at the cut surface without considering margin width. Additionally, in all cases, parenchymal transection was performed using the cavitron ultrasonic surgical aspirator, which is known to ablate or aspirate parenchyma along the transection plane [36, 37]. Therefore, there may be a certain number of patients who were inappropriately categorized on the basis of surgical margin status and our definition may have failed to stratify R0 and R1 resection, especially in patients with synchronous metastasis.

The present study has several limitations. First, the data were retrospectively collected from the database at a single center and comprised a small sample size. Second, the background data of the patients were not synchronized, indicating that selection bias may exist and affect the RFS and OS. Third, while the mutation status of *KRAS* was assessed, the differential effect of codons 12 and 13 was not considered. Moreover, somatic mutations in other genes such as *NRAS* and *BRAF* were examined only in a few patients and were not analyzed in the present study. Therefore, further studies should be conducted to assess the relationship of somatic mutations in multiple genes, such as *KRAS*, *NRAS*, and *BRAF,* with the clinical outcome of patients with CRLM.

## Conclusion

In conclusion, our study found *KRAS* mutational status as an independent prognostic factor in patients with synchronous CRLM, but not in those with metachronous CRLM. We propose that the treatment strategy should be planned on the basis of the timing of metastasis, considering its influence on the biological behavior of CRLM.

## Data Availability

The datasets used during this study are available from the corresponding author on reasonable request.

## References

[1] Zaydfudim VM, McMurry TL, Harrigan AM, Friel CM, Stukenborg GJ, Bauer TW, Adams RB, Hedrick TL (2015). Improving treatment and survival: a population-based study of current outcomes after a hepatic resection in patients with metastatic colorectal cancer. HPB (Oxford)..

[2] Engstrand J, Nilsson H, Stromberg C, Jonas E, Freedman J (2018). Colorectal cancer liver metastases - a population-based study on incidence, management and survival. BMC Cancer.

[3] House MG, Ito H, Gonen M, Fong Y, Allen PJ, DeMatteo RP, Brennan MF, Blumgart LH, Jarnagin WR, D'Angelica MI (2010). Survival after hepatic resection for metastatic colorectal cancer: trends in outcomes for 1,600 patients during two decades at a single institution. J Am Coll Surg.

[4] Pawlik TM, Schulick RD, Choti MA (2008). Expanding criteria for resectability of colorectal liver metastases. Oncologist..

[5] Elias D, Sideris L, Pocard M, Ouellet JF, Boige V, Lasser P, Pignon JP, Ducreux M (2004). Results of R0 resection for colorectal liver metastases associated with extrahepatic disease. Ann Surg Oncol.

[6] Leung U, Gonen M, Allen PJ, Kingham TP, RP DM, Jarnagin WR, et al. Colorectal cancer liver metastases and concurrent extrahepatic disease treated with resection. Ann Surg. 2017;265(1):158–65. 10.1097/SLA.0000000000001624. 10.1097/SLA.0000000000001624 PMC4942404 28009741

[7] Sawada Y, Sahara K, Endo I, Sakamoto K, Honda G, Beppu T, Kotake K, Yamamoto M, Takahashi K, Hasegawa K, Itabashi M, Hashiguchi Y, Kotera Y, Kobayashi S, Yamaguchi T, Tabuchi K, Kobayashi H, Yamaguchi K, Morita S, Natsume S, Miyazaki M, Sugihara K (2020). Long-term outcome of liver resection for colorectal metastases in the presence of extrahepatic disease: a multi-institutional Japanese study. J Hepatobiliary Pancreat Sci.

[8] Elias D, Cavalcanti A, Sabourin JC, Lassau N, Pignon JP, Ducreux M, Coyle C, Lasser P (1998). Resection of liver metastases from colorectal cancer: the real impact of the surgical margin. Eur J Surg Oncol.

[9] Adams RB, Langer B (1996). Resection margins for colorectal metastases to the liver: do they make a difference?. HPB Surg.

[10] Lordan JT, Karanjia ND (2008). Size of surgical margin does not influence recurrence rates after curative liver resection for colorectal cancer liver metastases (Br J Surg 2007; 94: 1133-1138). Br J Surg.

[11] Muratore A, Ribero D, Zimmitti G, Mellano A, Langella S, Capussotti L (2010). Resection margin and recurrence-free survival after liver resection of colorectal metastases. Ann Surg Oncol.

[12] Liu W, Zhou JG, Sun Y, Zhang L, Xing BC. The role of neoadjuvant chemotherapy for resectable colorectal liver metastases: a systematic review and meta-analysis. Oncotarget. 2016;7(24):37277–87. 10.18632/oncotarget.8671. 10.18632/oncotarget.8671 PMC5095075 27074564

[13] Nordlinger B, Sorbye H, Glimelius B, Poston GJ, Schlag PM, Rougier P, Bechstein WO, Primrose JN, Walpole ET, Finch-Jones M, Jaeck D, Mirza D, Parks RW, Collette L, Praet M, Bethe U, van Cutsem E, Scheithauer W, Gruenberger T, EORTC Gastro-Intestinal Tract Cancer Group, Cancer Research UK, Arbeitsgruppe Lebermetastasen und-tumoren in der Chirurgischen Arbeitsgemeinschaft Onkologie (ALM-CAO), Australasian Gastro-Intestinal Trials Group (AGITG), Fédération Francophone de Cancérologie Digestive (FFCD) (2008). Perioperative chemotherapy with FOLFOX4 and surgery versus surgery alone for resectable liver metastases from colorectal cancer (EORTC intergroup trial 40983): a randomised controlled trial. Lancet.

[14] Folprecht G, Gruenberger T, Bechstein WO, Raab HR, Lordick F, Hartmann JT, Lang H, Frilling A, Stoehlmacher J, Weitz J, Konopke R, Stroszczynski C, Liersch T, Ockert D, Herrmann T, Goekkurt E, Parisi F, Köhne CH (2010). Tumour response and secondary resectability of colorectal liver metastases following neoadjuvant chemotherapy with cetuximab: the CELIM randomised phase 2 trial. Lancet Oncol.

[15] Nordlinger B, Van Cutsem E, Gruenberger T, Glimelius B, Poston G, Rougier P, Sobrero A, Ychou M (2009). European colorectal metastases treatment G, sixth international colorectal liver metastases W. combination of surgery and chemotherapy and the role of targeted agents in the treatment of patients with colorectal liver metastases: recommendations from an expert panel. Ann Oncol.

[16] Tsai MS, Su YH, Ho MC, Liang JT, Chen TP, Lai HS, Lee PH (2007). Clinicopathological features and prognosis in resectable synchronous and metachronous colorectal liver metastasis. Ann Surg Oncol.

[17] Kobayashi S, Beppu T, Honda G, Yamamoto M, Takahashi K, Endo I, Hasegawa K, Kotake K, Itabashi M, Hashiguchi Y, Kotera Y, Sakamoto K, Yamaguchi T, Morita S, Tabuchi K, Miyazaki M, Sugihara K (2020). Survival benefit of and indications for adjuvant chemotherapy for resected colorectal liver metastases-a Japanese Nationwide survey. J Gastrointest Surg.

[18] Hasegawa K, Saiura A, Takayama T, Miyagawa S, Yamamoto J, Ijichi M, Teruya M, Yoshimi F, Kawasaki S, Koyama H, Oba M, Takahashi M, Mizunuma N, Matsuyama Y, Watanabe T, Makuuchi M, Kokudo N (2016). Adjuvant Oral uracil-Tegafur with Leucovorin for colorectal Cancer liver metastases: a randomized controlled trial. PLoS One.

[19] Nash GM, Gimbel M, Shia J, Nathanson DR, Ndubuisi MI, Zeng ZS, Kemeny N, Paty PB (2010). KRAS mutation correlates with accelerated metastatic progression in patients with colorectal liver metastases. Ann Surg Oncol.

[20] Karagkounis G, Torbenson MS, Daniel HD, Azad NS, Diaz LA, Donehower RC, Hirose K, Ahuja N, Pawlik TM, Choti MA (2013). Incidence and prognostic impact of KRAS and BRAF mutation in patients undergoing liver surgery for colorectal metastases. Cancer.

[21] Vauthey JN, Zimmitti G, Kopetz SE, Shindoh J, Chen SS, Andreou A, Curley SA, Aloia TA, Maru DM (2013). RAS mutation status predicts survival and patterns of recurrence in patients undergoing hepatectomy for colorectal liver metastases. Ann Surg.

[22] Schirripa M, Cremolini C, Loupakis F, Morvillo M, Bergamo F, Zoratto F, Salvatore L, Antoniotti C, Marmorino F, Sensi E, Lupi C, Fontanini G, Gregorio VD, Giannini R, Basolo F, Masi G, Falcone A (2015). Role of NRAS mutations as prognostic and predictive markers in metastatic colorectal cancer. Int J Cancer.

[23] Schirripa M, Bergamo F, Cremolini C, Casagrande M, Lonardi S, Aprile G, Yang D, Marmorino F, Pasquini G, Sensi E, Lupi C, de Maglio G, Borrelli N, Pizzolitto S, Fasola G, Bertorelle R, Rugge M, Fontanini G, Zagonel V, Loupakis F, Falcone A (2015). BRAF and RAS mutations as prognostic factors in metastatic colorectal cancer patients undergoing liver resection. Br J Cancer.

[24] Renaud S, Romain B, Falcoz PE, Olland A, Santelmo N, Brigand C, Rohr S, Guenot D, Massard G (2015). KRAS and BRAF mutations are prognostic biomarkers in patients undergoing lung metastasectomy of colorectal cancer. Br J Cancer.

[25] Takahashi M, Hasegawa K, Oba M, Aoki T, Sakamoto Y, Sugawara Y, Kokudo N (2015). Repeat resection leads to long-term survival: analysis of 10-year follow-up of patients with colorectal liver metastases. Am J Surg.

[26] Adam R, Bismuth H, Castaing D, Waechter F, Navarro F, Abascal A, Majno P, Engerran L (1997). Repeat hepatectomy for colorectal liver metastases. Ann Surg.

[27] Oba M, Hasegawa K, Matsuyama Y, Shindoh J, Mise Y, Aoki T, Sakamoto Y, Sugawara Y, Makuuchi M, Kokudo N (2014). Discrepancy between recurrence-free survival and overall survival in patients with resectable colorectal liver metastases: a potential surrogate endpoint for time to surgical failure. Ann Surg Oncol.

[28] Bellier J, De Wolf J, Hebbar M, Amrani ME, Desauw C, Leteurtre E, Pruvot FR, Porte H, Truant S (2018). Repeated resections of hepatic and pulmonary metastases from colorectal Cancer provide long-term survival. World J Surg.

[29] Mise Y, Zimmitti G, Shindoh J, Kopetz S, Loyer EM, Andreou A, Cooper AB, Kaur H, Aloia TA, Maru DM, Vauthey JN (2015). RAS mutations predict radiologic and pathologic response in patients treated with chemotherapy before resection of colorectal liver metastases. Ann Surg Oncol.

[30] Garcia-Carbonero N, Martinez-Useros J, Li W, Orta A, Perez N, Carames C, et al. KRAS and BRAF mutations as prognostic and predictive biomarkers for standard chemotherapy response in metastatic colorectal cancer: a single institutional study. Cells. 2020;9(1):219. 10.3390/cells9010219. 10.3390/cells9010219 PMC7016634 31952366

[31] Margonis GA, Amini N, Andreatos N, Sasaki K, McVey J, Mirza MB, Warner S, Buettner S, Barbon C, Wang J, Pulvirenti A, Angelou A, Kamphues C, Antoniou E, Pikoulis E, Pawlik TM, Kaczirek K, Poultsides G, Wagner D, Endo I, Imai K, Aucejo F, Kreis ME, Wolfgang CL, Weiss MJ (2019). KRAS mutational status impacts pathologic response to pre-hepatectomy chemotherapy: a study from the international genetic consortium for liver metastases. HPB (Oxford).

[32] Kobayashi S, Beppu T, Honda G, Yamamoto M, Takahashi K, Endo I, et al. Survival benefit of and indications for adjuvant chemotherapy for resected colorectal liver metastases-a Japanese Nationwide survey. J Gastrointest Surg. 2019. 10.1007/s11605-019-04250-9 31197683

[33] Margonis GA, Sasaki K, Andreatos N, Kim Y, Merath K, Wagner D, Wilson A, Buettner S, Amini N, Antoniou E, Pawlik TM (2017). KRAS mutation status dictates optimal surgical margin width in patients undergoing resection of colorectal liver metastases. Ann Surg Oncol.

[34] Brudvik KW, Mise Y, Chung MH, Chun YS, Kopetz SE, Passot G, Conrad C, Maru DM, Aloia TA, Vauthey JN (2016). RAS mutation predicts positive resection margins and narrower resection margins in patients undergoing resection of colorectal liver metastases. Ann Surg Oncol.

[35] Zhang Q, Peng J, Ye M, Weng W, Tan C, Ni S, Huang D, Sheng W, Wang L (2020). KRAS mutation predicted more Mirometastases and closer resection margins in patients with colorectal Cancer liver metastases. Ann Surg Oncol.

[36] Nuzzo G, Giuliante F, Ardito F, Vellone M, Giovannini I, Federico B, Vecchio FM (2008). Influence of surgical margin on type of recurrence after liver resection for colorectal metastases: a single-center experience. Surgery.

[37] Xu D, Wang HW, Yan XL, Li J, Wang K, Xing BC (2019). Sub-millimeter surgical margin is acceptable in patients with good tumor biology after liver resection for colorectal liver metastases. Eur J Surg Oncol.

